# Experiences from a cluster‐randomized trial (ParaNASPP) exploring triage and diagnostic accuracy in paramedic‐suspected stroke: a qualitative interview study

**DOI:** 10.1111/ene.16252

**Published:** 2024-02-25

**Authors:** Mona Guterud, Camilla Hardeland, Helge Fagerheim Bugge, Else Charlotte Sandset, Edel Jannecke Svendsen, Maren Ranhoff Hov

**Affiliations:** ^1^ Department of Research Norwegian Air Ambulance Foundation Oslo Norway; ^2^ Division of Prehospital Services Oslo University Hospital Oslo Norway; ^3^ Institute of Clinical Medicine University of Oslo Oslo Norway; ^4^ Department of Nursing, Health and Laboratory Science Østfold University College Halden Norway; ^5^ Department of Neurology Oslo University Hospital Oslo Norway; ^6^ Institute of Nursing and Health Promotion Oslo Metropolitan University Oslo Norway; ^7^ Department of Research Sunnaas Rehabilitation Hospital Oslo Norway

**Keywords:** ambulance, diagnostic accuracy, NIHSS, paramedic, prehospital, stroke

## Abstract

**Background and purpose:**

Timely prehospital stroke recognition was explored in the Paramedic Norwegian Acute Stroke Prehospital Project (ParaNASPP) by implementation of stroke education for paramedics and use of the National Institutes of Health Stroke Scale (NIHSS) through a mobile application. The study tested triage and facilitated communication between paramedics and stroke physicians. To complement the quantitative results of the clinical trial, a qualitative approach was used to identify factors that influence triage decisions and diagnostic accuracy in prehospital stroke recognition experienced by paramedics and stroke physicians.

**Method:**

Semi‐structured qualitative individual interviews were performed following an interview guide. Informants were recruited from the enrolled paramedics and stroke physicians who participated in the ParaNASPP trial from Oslo University Hospital. Interviews were audio recorded, transcribed verbatim and approached inductively using the principles of thematic analysis.

**Results:**

Fourteen interviews were conducted, with seven paramedics and seven stroke physicians. Across both groups two overarching themes were identified related to triage decisions and diagnostic accuracy in prehospital stroke recognition: prehospital NIHSS reliably improves clinical assessment and communication quality; overtriage is widely accepted whilst undertriage is not.

**Conclusion:**

Paramedics and stroke physicians described how prehospital NIHSS improved communication quality and reliably improved prehospital clinical assessment. The qualitative results support a rationale of an application algorithm to decide which NIHSS items should prompt immediate prenotification rather than a complete NIHSS as default.

## BACKGROUND

After the initial call to an Emergency Medical Communication Centre paramedics are the next to assess a patient with suspected stroke [1, 2], and competence in symptom recognition is crucial for rapid and correct triage to time‐sensitive treatment [1, 3]. A 30% overtriage, when screening stroke patients in the prehospital setting, is recommended to avoid missing patients suffering an acute stroke [4, 5]. Further overtriage will strain scarce resources and may delay appropriate treatment for time‐sensitive stroke mimic conditions.

Prehospital stroke scales originate from the in‐hospital National Institutes of Health Stroke Scale (NIHSS) [2], although the terminology differs. Most prehospital stroke scales are designed to detect large vessel occlusions in the anterior circulation and primarily focus on classic stroke symptoms, such as the Face–Arm–Speech–Time (FAST) test [2]. The FAST is the stroke scale of choice in Norwegian prehospital protocols, although data from the Norwegian stroke registry show that only 70% of stroke patients have a FAST symptom [6]. Studies on prehospital stroke identification show that approximately 30% are not identified [7, 8], indicative of a highly problematic undertriage. NIHSS is a broader stroke scale that captures a more heterogeneous stroke population [9] but has been regarded as too complex and time consuming for prehospital use [10, 11]. This assumption has been challenged in studies demonstrating reliable use of NIHSS by trained paramedics [12, 13, 14].

Interprofessional differences in training and communication styles increase the risk of misdiagnosis and delayed treatment for patients [15], and effective communication is a prominent factor for streamlining clinical pathways [16]. The lack of a common language has been identified as a major challenge when prehospital and in‐hospital teams collaborate [17]. Training and standardized tools may improve interprofessional communication [15]. The utilization of different stroke scales is a communication barrier as they do not provide a common language between the prehospital and in‐hospital teams [12, 18]. Low quality communication between prehospital and in‐hospital teams regarding stroke patients significantly increased the prehospital on‐scene time in a Danish study [19]. Still, the establishment of a common language is missing in stroke care. Despite recommendations for training paramedics in stroke recognition, the use of prehospital stroke scales and a prehospital notification [1, 3], there is scarce evidence on the type of training, favoured prehospital stroke scale or prenotification quality.

The Paramedic Norwegian Acute Stroke Prehospital Project (ParaNASPP) clinical trial assessed whether the eSTROKE intervention of a mobile application with NIHSS to communicate and transfer the results of the prehospital assessment to the stroke physicians could improve triage and diagnostic accuracy of acute stroke patients (Appendix S3). The control was a standard paramedic stroke protocol consisting of FAST and ordinary telephone communication with the stroke physician for triage decisions [20]. The intervention did not increase the positive predictive value for final diagnosis of stroke and overtriage remained high, meaning that a large proportion of patients admitted with suspected acute stroke did not have a final diagnosis of stroke. However, patients in the intervention group had lower NIHSS scores at admission, indicating reduced undertriage since subtler strokes were identified. Compared to the control group, the intervention group had significantly longer prehospital time, whilst in‐hospital time from arrival to first computer tomography and treatment decision was significantly shorter. Time from onset to thrombolysis was significantly longer in the intervention group, possibly related to subtler strokes requiring more deliberation in examination and triage decisions throughout the entire stroke chain. To further understand these results and add to the knowledge, a qualitative extension was used to explore real‐life trial experiences from paramedics and stroke physicians [21, 22, 23]. This study aimed to identify factors influencing triage decisions and diagnostic accuracy in prehospital stroke recognition experienced by paramedics and stroke physicians.

## METHODS

The ParaNASPP trial originated from two other stroke studies in Norway [24, 25] and was a stepped‐wedge cluster‐randomized controlled trial that took place at Oslo University Hospital (OUH) [16]. The paramedics received stroke education through an e‐learning platform, followed by a practical day of training supervised by experienced stroke physicians. During the trial paramedics used the eSTROKE application to perform NIHSS on patients with suspected acute stroke and to consult with the stroke physicians. Results from the prehospital examination were transferred directly to an in‐hospital version of the application on the stroke physician's phone and enhanced the prenotification made to the in‐hospital team. A more detailed description of the method in the clinical trial is published elsewhere [26].

### Qualitative interviews

The qualitative method is elaborated in Appendix S1. Two months after the clinical trial was completed participants were invited to be interviewed. Our interest was in the specific experiences with the intervention, and a purposive sampling strategy was applied [27]. Volunteers were recruited from the 267 trained paramedics through the ambulance services' information channels. Stroke physicians employed at the Stroke Unit, OUH, were recruited with a direct request. The only criterion set for the informants was trial participation. All volunteers were interviewed and included in the analysis.

Two pilot interviews resulted in a revised interview guide (Appendix S1). Researchers not included in the clinical trial performed the interviews and participated throughout the analysis process. Twelve of 14 interviews were performed prior to presentation of the clinical trial main results. All interviews were performed digitally; audio was recorded and stored at a secure server. All data were transcribed verbatim and de‐identified, resulting in 135 pages of transcribed text. Transcripts were inductively analysed in accordance with the principles of thematic analysis [28, 29]. An example of the analysis process is presented in a coding tree (Table 1). Consensus was not a primary aim for the joint analysis process but an effort for shared reflection to broaden perspectives [29]. For writing this paper, the COnsolidated criteria for REporting Qualitative research (COREQ) checklist (Appendix S2) was consulted [30]. Any identifying attributes have been removed and, to display variety in the data, participants are referred to as P(X) for the paramedics or S(X) for the stroke physicians. A description of the participants is found in Table 2, displaying a broad distribution of attributes comparable to a real‐world setting, except a lower proportion of females.

**TABLE 1:** Description of coding tree.

Raw data	Meaning bearing content	Code	Subtheme	Main theme
Before, most of the paramedics would do the same as anyone on the street, and that made us ask for more information, like what are really the symptoms. You had to, yes, you spent many more resources during the phone call, to do the examination via the paramedic and it was like a whispering game and where the paramedic not always understood what you meant	The lack of common ground is time‐consuming and creates misunderstanding	More standardized and efficient consultations	A common language supports decision making and is time‐efficient	Prehospital NIHSS reliably improves clinical assessment and communication quality

**TABLE 2:** Interview participant demographics.

	(*n* = 14)
Age	
<39	10
>40	4
Sex	
Female	4
Male	10
Years of experience	
<5	4
6–10	3
>10	7
	(*n* = 7)
Educational level prehospital	
Emergency Medical Technician	3
Paramedic	4
	(*n* = 7)
Educational level in‐hospital	
Geriatric registrar	2
Neurology registrar	1
Neurology consultant	2
Geriatric consultant	2

Quotations are adjusted if necessary for translation purposes. Further, square brackets are used if an explanation is necessary and round brackets indicate selected pieces from the transcription.

The participants consented verbally at the time for the interview and a written informed consent was delivered either prior to or after completion of the interview session. The Regional Committee for Medical Research Ethics Southeast Norway approved the clinical trial (2018/2310), and the local data protection office at OUH approved the qualitative study (21/23988).

## RESULTS

Throughout the data analysis two overarching themes were identified with subthemes related to triage decisions and diagnostic accuracy in prehospital stroke recognition (Table 3). Examples of quotations from the interviews are presented in Table 4.

**TABLE 3:** Themes and subthemes identified.

Themes	Subthemes
Prehospital NIHSS reliably improves clinical assessment and communication quality	NIHSS provides standardized and valuable information in the prehospital setting A common language supports decision making and is time‐efficient
Overtriage is widely accepted whilst undertriage is not	Prehospital aspects and stroke characteristics are inherent barriers for diagnostic accuracy Concurrent circumstances counteract diagnostic accuracy

**TABLE 4:** Example quotations from the interviews.

NIHSS provides standardized and valuable information in the prehospital setting	P7: FAST is a great tool, but doesn't cover enough (…) to confidently rule out stroke (…) it is very useful when you can visibly detect stroke, more or less. Then you use FAST. But NIHSS is more comprehensive (…) it has given me much more knowledge about that type of stroke we almost never managed to identify before P6: One thing I noticed about FAST was that many [paramedics] felt it was a bit too simple. I've encountered several colleagues who picked up a few tests from observing neurologists perform a NIHSS examination at the hospital. From there, they [the paramedics] learned a lot of different things. I think there are also many who don't quite understand what they've examined P4: If I come across a patient with very clear symptoms (…) I wouldn't use the app [to score NIHSS] S4: [About NIHSS] I get a more detailed picture now. I know that the paramedics have performed a detailed examination (…) I find it much easier to trust what they say when they call me S6: Even though we weren't always necessarily in agreement about the scoring, one has thought the same thoughts, structured them and made the same assessments, even if the result of the assessment might not necessarily be the same S3: For example, with NIHSS, I've received incorrect information. That happens often with paresis and ataxia, for instance. That ataxia is difficult to assess (…) So often when it's ataxia (…) and when they come in, it's paresis (…) But this also applies to my colleagues in the stroke unit. Because it's, yes, how people interpret it S3: If (…)[the paramedic] see that the patient has clear symptoms, there's no need to inquire into further diagnostics regarding that. Instead, place a green IV line so they [the patients] are ready when they arrive S6: I think NIHSS is a pretty good assessment overall for stroke. FAST is very imprecise. You can have quite significant neurological deficits that aren't captured in a FAST examination (…) I would choose NIHSS every single day S4: ParaNASPP has made paramedics much more attentive to what might be a stroke. I believe, and that's what I'm looking forward to see in the statistics, right, do we find more strokes? I think we are because it has become a much more structured examination S5: [The paramedics] (…) identify strokes from the posterior circulation, or strokes with symptoms that are not classic (…) I think this is the biggest and clearly the most important difference S5: [About consultation before] They [the paramedics] say it's just something odd, but they don't know what it is. He [the patient] has good strength in his arms and legs (…) something doesn't add up (…) There's something off here. But (…) [now] had gone through it systematically (…) the patient actually has significant neglect and visual field deficits. Extremely useful
A common language supports decision making and is time‐efficient	P7: Before ParaNASPP (…) when I called the stroke physician (…) we had a language problem because I was unable to describe what I saw (…) and the stroke physician wouldn't understand what I meant, so I spent more time (…) now we have the same good communication with the stroke physician as with a cardiologist (…) that has been a major change for me P4: When they [the stroke physicians] meet us, they have more information [about the patient], and we don't have to repeat ourselves so often, in the report P7: I feel that they [the stroke physicians] trust that we know what we're doing. And everything goes a lot faster this way, I think. And I feel in control. I know what I'm doing! S5: Well, I have to say that initially, I might have been a bit sceptical because how much time should one spend outside if you're sure this is a stroke? (…) But, well, I think that scepticism actually disappeared quite quickly. You become so happy when you can have a much more focused communication and talk about it, understanding each other better then. Talk about the same thing. Right? S4: (…) that we can share the results (…) at the same time as they [the paramedics] call us, that we can discuss NIHSS, this amplifies that we are talking about the same things (…) we have, sort of, the same picture of the patient S7: Because we feel (…) a lot more prepared and we can speed up things in hospital with the NIHSS score available [in the application] before the patient arrives [to the emergency room] S4: [Before] most of the paramedics would do the same as anyone on the street, and that made us ask for more information (…) what are really the symptoms. You had to, yes, you spent much more resources during the phone call, to do the examination via the paramedic (…) like a whispering game (…) where the paramedic not always understood what you meant
Prehospital aspects and stroke characteristics are inherent barriers for diagnostic accuracy	P2: Well, there are all these differential diagnoses. It could be that older patients experience stroke‐like symptoms from having a full bladder and urinary retention, you know. But, we don't have a bladder scanner (…) But there are many such things, you know (…) And then, it's difficult to determine if it's a stroke or something else, especially if it's hard to communicate with the patient, of course. (…) Our patients aren't in a hospital room where everything is organized. Our patients are under the sofa, behind the bed, squeezed between the shower and the toilet. There are so many other factors and the overall picture that can make it harder to detect a stroke S5: And it's a bit like, we're terrified of undertriaging in a way. That's when it potentially can be very harmful for the patient. That's why, in a way, we're quite on edge (…) there's a low threshold to see the patient quickly and alert the [stroke] team S4: They [the paramedics] have their findings, then the patient arrives at the hospital, and we quickly realize that this was nothing. But we also have a completely different set of diagnostic tools, right? Examinations and things we can do in the hospital. So it's much easier to say things with certainty after an hour in the ED than 10 min at the patient's home. There's a great deal of humility for that S2: Sometimes the symptoms are so diffuse that, well, it's difficult to reject the patient even if we don't believe it's a stroke S4: (…) that with ParaNASPP and NIHSS you get a good assessment of the acute situation and the picture that is, but it can also act as a lever, so to speak, to push the patient into acute assessment (…) to say that this *is* a symptom of a stroke S5: I understand that sometimes the patient is so unwell that they [the paramedics] just want to get the patient hospitalized (…) those are the times when the triaging has gone wrong (…) I think all parties panic a bit because the patient is so unwell, and it's not entirely clearly communicated what the patient's actual problem is
Concurrent circumstances counteract diagnostic accuracy	P7: When we (…) arrive at the patient (…) we think worst‐case scenario and sort of start from there. What is the worst thing that could be wrong with this patient here and now. And then we start from that point P2: Sometimes you just have to call to get help (…) if it's difficult to assess the patient. If it's challenging to perform NIHSS I'll call and ask: Do you want to look at this yourself (…) and try to conduct a proper examination? Yes. I call them [the stroke physician] when I'm convinced, but I also call when I want a sparring partner P1: Even if the suspicion of stroke is quite low (…) I still may choose to consult with the stroke physician to access more information S5: And especially when there's a lot to do (…) it's obvious that communication becomes very limited (…) and when you're pressed on time, that will probably lead to overtriage to a greater extent than undertriage S6: [The paramedics] lack the differential diagnostic capacity that one might have in a hospital setting. In that sense, it can create tunnel vision blocking other diagnostics. A ParaNASPP‐trained paramedic (…) might identify a stroke in situations where it necessarily isn't a stroke. It's like searching, finding what one is looking for, yes (…) and to the paramedics' defence as well, difficult diagnostic assessments have been greatly influenced by a preconceived stroke‐centric mindset S2: We are often conferred about patients who are confused (…) which isn't necessarily a stroke (…) in very few cases, it relates to a stroke (…) And syncope. I think we receive quite a few requests about these two conditions. They [the paramedics] first contact us, then (…) other specialists

Abbreviations: ED, emergency department; FAST, Face–Arm–Speech–Time; NIHSS, National Institutes of Health Stroke Scale; ParaNASPP, Paramedic Norwegian Acute Stroke Prehospital Project; P(X), paramedic; S(X), stroke physician.

### Prehospital NIHSS reliably improves clinical assessment and communication quality

Overall, the paramedics and stroke physicians experienced that the NIHSS improved the paramedics' clinical assessment of acute stroke. Prehospital NIHSS created a common language between paramedics and stroke physicians. The participants reported the consultation to be more informative and time‐efficient. Two subthemes further described the effect of NIHSS and communication benefits.

#### NIHSS provides standardized and valuable information in the prehospital setting

The FAST test was often found too superficial, and before the trial the paramedics experienced that they fell short on competence and skills. The paramedics had recognized this shortcoming and pursued knowledge by copying stroke physicians or seeking literature on their own, leading to non‐standardized assessments. Both groups reported how the intervention with NIHSS had extended and enhanced the prehospital assessment, and that it was beneficial for identifying a broader spectrum of neurological deficits. Still, a complete NIHSS was sometimes found too detailed in assessment of classic stroke symptoms, and both groups then perceived FAST as accurate enough. Paramedics described how they would use their clinical judgement to opt out of a complete NIHSS when severe classic stroke symptoms were present. Further, they mentioned the complexity of NIHSS and a need to practise and refine, as items like ataxia and neglect were difficult and prone to subjective interpretation. The stroke physicians also recognized this but pointed out that this was not a distinct prehospital challenge. Regardless, the stroke physicians embraced the use of prehospital NIHSS. They pointed out how FAST is not readily compatible with the in‐hospital scale and assessment; hence they were better informed with prehospital NIHSS. In cases where the prehospital NIHSS turned out be inaccurate or questionable, the stroke physicians were still more prepared on patient status and valued the possibility for evaluating trends in the patient's NIHSS score.

The paramedics described being dispatched to numerous suspected acute stroke incidents that after examination no longer warranted a suspicion of acute stroke. After the intervention, paramedics felt more confident when stroke suspicion was both sustained or dismissed after assessment. The increased competence and the broader examination were experienced as a support for both leaving a patient on‐scene or pursuing other explanations for symptom presentation.

#### A common language supports decision making and is time‐efficient

Both paramedics and stroke physicians reported that communication was easy, trustworthy and efficient with prehospital NIHSS. Prior to the intervention, the paramedics felt they lacked the vocabulary to fully describe observations. Based on clinical judgement formed by experience and intuitive perception they sensed that they missed important findings. The NIHSS provided the tool to identify these and the confidence and language to communicate their findings. For the stroke physicians, prehospital NIHSS gave a new tool for making decisions on the level of treatment for consulted patients with less back‐and‐forth communication and reduced the chance of misinterpretations.

The stroke physicians reported that the visualization of the prehospital assessment parallel to the verbal communication added extra benefits to the consultation and perceived this to reduce in‐hospital delay. The paramedics underlined fewer misunderstandings since the history and examinations were standardized.

### Overtriage is widely accepted whilst undertriage is not

Both stroke physicians and paramedics presented multiple justifications for a deliberate choice to overtriage and the challenges in balancing the necessary and acceptable overtriage against the concern for undertriage. Several of the examples mentioned were linked to structural qualities of the prehospital area and stroke itself, whilst others referred to organizational considerations or concurrent conditions. Two subthemes were identified describing different challenges with mistriage and thereby diagnostic accuracy.

#### Prehospital aspects and stroke characteristics are inherent barriers for diagnostic accuracy

That acute stroke may present itself over a broad and diverse symptom spectrum was reported to be particularly challenging for prehospital stroke recognition and triage decisions, especially in combination with the heterogeneous patient population. This was further complicated by inherent qualities of the prehospital setting such as information shortage and expectations of time‐efficiency. The paramedics stated that they were comfortable with overtriage if at all in doubt. This led to frequent consultations with the stroke physicians, sometimes as a strict precaution and at other times to access an important source of knowledge and discuss with a more highly qualified colleague. The stroke physicians underlined the nonspecific symptom presentation and a lack of possibilities to rule out stroke in the prehospital setting as acceptable reasons for overtriage, expressing a concern for undertriage. They also found it difficult, at times, to be the one responsible for saying no, even in cases of low suspicion of acute stroke.

#### Concurrent circumstances counteract diagnostic accuracy

The paramedics described how dispatches with a vague label of ‘something cerebral’ had increased in recent years. The paramedics interpreted these dispatches as possible stroke, but they often turned out to be patients with various intoxications. The paramedics reported this practice to increase the risk of them being less focused when responding to ‘something cerebral’. Implicit and explicit expectations of efficiency worsened these situations and increased the risk for both undertriage and overtriage. In general, the paramedics adhered to an unwritten rule of always assuming the most serious diagnosis until proven otherwise and assessing for acute stroke would often be a starting point. In addition to a responsibility to act as the patient's advocate, low organizational support was referred to as an argument for overtriage. Consequently, cases with low stroke suspicion would still end in a consultation with the stroke physician. The stroke physicians were to some degree familiar with this strategy and understood the paramedics' position. Sometimes it was easier and quicker to accept a patient for assessment, confirming the low threshold for in‐hospital acute assessment, especially when the in‐hospital workload was high. This was explained by their fear of missing out on important information in a consultation characterized by stress and heavy work pressure. Both groups reflected upon the increased focus on stroke detection, although different perspectives were present. The paramedics considered the trial to be a major contribution for a long‐awaited stroke education, the NIHSS to be a valuable tool for neurological examination, and that their competence in evaluation of stroke‐like symptoms had increased. A few of the stroke physicians experienced prehospital assessment with NIHSS when not indicated. Concerns about a liberal and arbitrary use of NIHSS were raised; however, NIHSS was preferred over FAST.

## DISCUSSION

The present study aimed to identify factors that influence triage decisions and diagnostic accuracy in prehospital stroke recognition experienced by paramedics and stroke physicians. Our qualitative results complement the results of the clinical trial and add important knowledge of prehospital stroke recognition and diagnostic accuracy in paramedic suspected stroke. Both paramedics and stroke physicians reported prehospital NIHSS to be time‐efficient and helpful in decision making by including more information in the assessment and to the communication. With a more detailed examination and a common language to describe clinical stroke presentation, prehospital NIHSS was experienced to be especially beneficial for recognition and subsequently correct triage in situations where the paramedics had a stroke suspicion without classic stroke symptoms. Both groups had a clear impression of reduced undertriage with the intervention, whilst overtriage was seen as unavoidable and necessary, although the stroke physicians raised some concerns of unwarranted overtriage. Different structural and contextual aspects were identified to heavily influence the endeavours for diagnostic accuracy.

Prenotification is important but compliance is known to vary [31]. Inability to directly communicate with the hospital has been identified as a major obstacle for prenotification to happen [32]. The standard OUH model already requires a consultation with the stroke physician prior to transportation to the hospital, and the eSTROKE intervention provided an enhanced version of this prenotification. The qualitative data support that the combination of NIHSS and transfer of prehospital data to the stroke physician improved prenotification quality by expanding the information exchange.

Recognition of strokes with classic stroke symptoms seems to be straightforward [8], but prehospital stroke tools like FAST may be too restrictive [18, 33] as 30% of acute strokes are not currently identified in the prehospital setting [6, 7, 8]. This demonstrates the importance of prehospital stroke scales to cover more than classic stroke symptoms. For example, NIHSS items like visual field and ataxia are important items to assess to increase sensitivity for posterior circulation stroke symptoms [34, 35]. Paramedics prefer the same scale as in‐hospital colleagues since identical examination elements improve communication [18], and a common language may reduce prehospital delay in stroke care [12, 18, 19]. Our participants recognized these benefits. The detection of more posterior circulation strokes was not confirmed in the clinical trial; however, there was an increased identification of strokes with a more subtle symptom presentation [20]. Timely stroke recognition and facilitated communication are reasonable arguments to pursue the implementation of prehospital NIHSS.

Our participants reported NIHSS to be time‐efficient for communication prior to hospitalization and how this effect extended after emergency department arrival. Of note, when symptoms were perceived to be clear‐cut, our participants found prehospital NIHSS to be less necessary and deliberately omitted the full examination. The need to minimize delay is indisputable [1, 3] but should include quality in triage decisions for all stroke patients. Prehospital stroke assessment with the NIHSS may not be more time consuming than with FAST [13]; still longer on‐scene time was found for the intervention group in the ParaNASPP trial [20]. Pandemic restrictions were argued to have confounded the prehospital time variables in the trial, and our participants experienced prehospital NIHSS to be time‐efficient, supporting this interpretation. The clinical judgement made by paramedics bypassing the intervention in clear‐cut cases was supported by the stroke physicians and adds to the rationale of an application algorithm to decide which NIHSS items should prompt immediate prenotification rather than a complete prehospital NIHSS as default [20].

Based on our qualitative data, explanations for overtriage in the clinical trial are multifactorial. Acute stroke constitutes 1%–2% of all Emergency Medical Communication Centre calls [36] and results in few verified stroke patients assessed by each paramedic, but many patients assessed with possible stroke symptoms. A high sensitivity for stroke recognition is set as a primary goal for dispatchers [37]; however, the absence of standardized competence and common language for acute stroke from dispatch make the increase in dispatches with ‘something cerebral’ likely to be an accurate observation made by the paramedics in our study. With suboptimal dispatcher stroke recognition and diagnostic accuracy [37] the paramedics meet an unselected patient population with a low stroke prevalence. In the ParaNASPP trial the positive predictive value for a final diagnosis of stroke was less than 50% in both the control and the intervention group; however, a more heterogeneous patient population was identified and conferred in the trial [20], and increased sensitivity for a final diagnosis of stroke is likely.

Despite the increased confidence in triage decisions reported by the paramedics, they described a deliberate practice of choosing caution over risk when considering stroke as diagnosis. Of note, this ambiguity was to some extent present in stroke physicians' descriptions as well. Paramedics are more concerned for undertriage than the consequences of overtriage, and risk management is tied to organizational policy as well as patient concern [38]. The wide spectrum of symptom presentation, high rates of stroke mimic conditions, time‐sensitivity and contextual challenges are all described by paramedics to impact stroke recognition [18, 38], consistent with our findings. The paramedics reported that rapid and correct identification of all possible stroke symptoms was a challenge in unclear situations. To overlook a stroke in the prehospital setting was considered blameworthy, and the paramedics could insist on a patient being accepted for acute stroke assessment. The stroke physicians reported difficulties in rejecting conferred patients, even in cases where stroke was an unlikely diagnosis, resulting in an unwarranted overtriage. The eSTROKE intervention did not sufficiently accommodate this, and alternatives that add to information availability should be explored. Telemedicine in stroke is promising [39, 40], but the paramedics' gatekeeper function requires a simultaneous focus on competence in stroke recognition to avoid overwhelming in‐hospital resources. Whether a video conference can reduce redundant overtriage in our setting should be explored. Based on the stroke physicians' reports of challenges with rejecting patients after being consulted, a video conference upon their initiative might add benefits to the prehospital assessment and voice call. The present study supports the use of prehospital NIHSS, but also identifies triage challenges not accommodated with the trial intervention. The future of prehospital stroke care should allow for a streamlined pathway for all stroke patients, and it is believed that the eSTROKE intervention with further development should be explored as one solution to achieve this.

Our study has several strengths and limitations. All apart from two interviews took place before the results of the clinical trial had been presented, limiting an effect of confirmation bias. Exploration of experiences from both the prehospital and in‐hospital trial participants is an additional strength [41]. Interviews conducted with volunteers may have recruited already positive participants and therefore biased the experiences. The same research group as in the clinical trial has conducted the present study. This may bias the interpretation of the qualitative data, and this limitation was reduced by using researchers with no participation in the clinical trial to conduct the interviews and to participate in the analysis process. NIHSS and prehospital notification to the stroke physician were conducted with a mobile application, limiting transferability to other regions as paramedic initiated transportation and a pre‐arrival notification of emergency department staff are more common than the OUH model with direct telephone contact. However, a smart phone application is accessible in most settings. Comparable standardized training models and customized methods for communication that consider local conditions and resources may enable implementation in other settings.

## CONCLUSION

Paramedics and stroke physicians described how prehospital NIHSS improved communication quality and reliably improved prehospital clinical assessment. They had a clear perception that more strokes were identified, and this supports an assumption of increased sensitivity for a broader part of the stroke population and reduced undertriage. Stroke characteristics, inherent prehospital limitations and fear of undertriage were explanations for an intentional willingness to overtriage. The qualitative results support a rationale of an application algorithm to decide which NIHSS items should prompt immediate prenotification rather than a complete NIHSS as default and should be explored in future studies.

## AUTHOR CONTRIBUTIONS

**Mona Guterud:** Writing – original draft; conceptualization; investigation; writing – review and editing; formal analysis; software; methodology. **Camilla Hardeland:** Investigation; methodology; writing – review and editing; formal analysis; software; validation. **Helge Fagerheim Bugge:** Writing – review and editing. **Else Charlotte Sandset:** Writing – review and editing. **Edel Jannecke Svendsen:** Investigation; writing – review and editing; validation; methodology; formal analysis. **Maren Ranhoff Hov:** Conceptualization; writing – review and editing; supervision.

## FUNDING INFORMATION

The study was funded by the Norwegian Air Ambulance Foundation, a non‐governmental organization for research and development.

## CONFLICT OF INTEREST STATEMENT

All authors declare no competing interests.

## ETHICS STATEMENT

The Regional Committee for Medical Research Ethics Southeast Norway approved the clinical trial (2018/2310). According to Norwegian laws and regulations, the local data protection office at Oslo University Hospital approved this qualitative study (21/23988).

## PATIENT AND PUBLIC INVOLVEMENT

The National Association for Heart and Lung disorders (LHL), a member‐based ideal organization, engaged in the protocol design for the clinical trial.

## Supporting information


Appendix S1

Appendix S2

Appendix S3

## Data Availability

Due to the nature of the qualitative data, this will not be made available.

## Interview guide paramedics

**Table:**

Themes	Research questions	Interview questions
Information on participant background		The following topics will be covered in the interview: Brief presentation of yourself Contextual conditions Which tools were previously used in stroke suspicion? Use of the new clinical tool Communication with stroke physician/paramedics before and after the intervention Perceived need for competence and perceived learning throughout the intervention period The reason why you have been invited is that you have been involved in the implementation of the clinical trial ParaNASPP which deals with the use of a new clinical tool when stroke is suspected Can you tell us a little about yourself Age Part‐time/full‐time Station affiliation Prehospital experience Level of education Other relevant work experience
Contextual conditions	Working conditions/psychosocial environment/other framework conditions at the ambulance service OUH? How can this affect communication in the acute stroke chain?	Can you describe a normal working day at the ambulance service in OUH? How do you perceive the staffing, the workload? How does workload affect the quality of communication with other collaborating partners in the prehospital chain? Can you tell us a little about your own motivation in relation to your job in the ambulance department? How can you develop professionally at your workplace? Other conditions you think influence your job as paramedic?
Clinical competence on stroke	Knowledge of stroke and stroke treatment Knowledge of symptoms and risk factors related to stroke What prevents or facilitates communication with a stroke physician in cases of suspected stroke? What factors are barriers to or facilitate communication between prehospital and in‐hospital teams in cases of suspected stroke	Now we are going to talk about recognizing patients with suspected stroke How do you think that you can contribute to the best possible healthcare for patients with suspected stroke? Before the trial, how did you communicate with the stroke physician when you suspected a stroke? Can you describe how you worked with the stroke physician during the trial? What makes it difficult to recognize stroke patients? In what situations do you contact your stroke doctor for consultation? When do you choose not to contact us if you have used the app?
Use of application and prehospital NIHSS	What influences the use of the application in patients with suspected stroke How does NIHSS influence your assessment of stroke symptoms when evaluating a patient with suspected stroke	Can you tell us a little about your experience with the training that was given in using the application? How do you think NIHSS serves as a tool in relation to FAST in prehospital assessment of suspected stroke? How does NIHSS influence communication with stroke doctors? In which situations do you use the application? Has the intervention changed the communication with the stroke physician? How? What has the usability been like for the app? Have you experienced that something is missing in the eSTROKEapp?
	Other important issues	Are there things we've forgotten to ask you about that you think are important for us to know?

## Interview guide stroke physicians

**Table:**

Theme	Research questions	Interview questions
Information on participant background		The following topics will be covered in the interview: Brief presentation of yourself Contextual conditions Which tools were previously used in stroke suspicion? Use of the new clinical tool Communication with stroke physician/paramedics before and after the intervention Perceived need for competence and perceived learning throughout the intervention period The reason why you have been invited is that you have been involved in the implementation of the clinical trial ParaNASPP which deals with the use of a new clinical tool when stroke is suspected Can you tell us a little about yourself Age Part‐time/full‐time Affiliation Experience Level of education Other relevant work experience
Contextual conditions	Working conditions/psychosocial environment/other framework conditions at the stroke unit at OUH? How can this influence communication in the acute stroke chain?	Can you describe a normal working day at the ambulance service in OUH? How do you perceive the staffing, the workload? How can you develop professionally at your workplace? How does workload whilst on duty influence communication with paramedics? How often? Can you say something about your motivation in relation to the job at the stroke unit? How can you develop professionally at your workplace? Other conditions you think influence your job as a stroke physician?
Clinical competence on stroke	Knowledge of stroke and stroke treatment Knowledge of symptoms and risk factors related to stroke What prevents or facilitates communication with a paramedic in cases of suspected stroke? What factors are barriers to or facilitate communication with paramedics in cases of suspected stroke?	Now we are going to talk about prehospital recognition of suspected stroke Before the trial, how did you communicate and collaborate with paramedics when they suspected a stroke? Can you describe how this was during the trial? What do you think is important that paramedics include in the consultation with a stroke physician? Optionally: What do you think a paramedic can contribute for the best possible healthcare for patients with suspected stroke? Can you tell us about when paramedics have made your job as a stroke physician easier in assessing patients with suspected stroke? Can you tell us about a time when communication with paramedics has complicated your job as a stroke physician in assessing patients with suspected stroke? What is underemphasized when paramedics contact a stroke physician? What is overemphasized when paramedics contact a stroke physician? What do you think needs to be done to improve the collaboration between you and the paramedics?
Use of application and prehospital NIHSS	What influences the use of the application in patients with suspected stroke How does NIHSS influence your assessment of stroke symptoms when evaluating a patient with suspected stroke	Can you tell us about your experience with the training that was given in using the application? What do you think about NIHSS as a tool compared to FAST in prehospital assessment of stroke suspicion? How does NIHSS simplify or complicate communication with paramedics? How do you prefer the assessment of patients with suspected stroke to be communicated? (NIHSS or FAST/App or not app) Has communication with paramedics changed since the intervention in the trial? How? How do you rate the user‐friendliness of the application? Do you think that something is missing in the application? What?
	Other important issues	Are there things we've forgotten to ask you about that you think are important for us to know?
