# Supplementary material for: Experiences from a cluster‐randomized trial (ParaNASPP) exploring triage and diagnostic accuracy in paramedic‐suspected stroke: a qualitative interview study
Source: Eur J Neurol. 2024 Feb 25;31(5):e16252. doi: 10.1111/ene.16252 (PMC11235795; doi:10.1111/ene.16252)
Supplement: Supplementary file 1 — Appendix S1 [file ENE-31-e16252-s002.docx]

**Elaboration of the qualitative method**

We offered participants one thousand NOK (€100) as a compensation for time spent in the interview. The interviews lasted from 22 minutes to 83 minutes, and 9 hours and 48 minutes in total. The interview guide was developed by the research team and evaluated in two pilot interviews, not included in the analysis. Two pilot interviews resulted in a revised interview guide. In total, fourteen interviews were conducted between 10 January 2022 and 30 May 2022. Twelve of fourteen interviews were performed prior to presentation of the clinical trial main results. All interviews were performed digitally on the videoconferencing platform Zoom. The interviewer and co-author (CH) had no prior relationship to the participants, or involvement in the clinical trial. Only audio was recorded and stored at a secure server. All data were transcribed verbatim and de-identified by a study nurse, resulting in 135 pages of transcribed text. Based on the rich descriptions from the interviews, we concluded that more interviews were unlikely to provide any substantial added information, in accordance with a realist approach to thematic analysis. Transcripts were inductively analysed in HyperRESEARCH, a qualitative data analysis software, in accordance with the principles of thematic analysis by Braun and Clarke. The analysis was a collaboration between MG who is a paramedic and PhD candidate in the ParaNASPP project, hence well-known by participants, and co-authors CH and EJS who are both nurses and associate professors with experience in qualitative research analysis. The analysis process started with familiarisation with the transcribed interviews by repeated reading and condensing of the data by coding and initial grouping (MG). In the next step we searched for and identified themes across the coded data set based on a straightforward interpretation of the data. Following that, we revisited the codes and searched for viable alternative themes in an iterative process, before we concluded (MG, CH, EJS).
