# Supplementary figures and images for: Experiences from a cluster‐randomized trial (ParaNASPP) exploring triage and diagnostic accuracy in paramedic‐suspected stroke: a qualitative interview study
Source: Eur J Neurol. 2024 Feb 25;31(5):e16252. doi: 10.1111/ene.16252 (PMC11235795; doi:10.1111/ene.16252)

## The ParaNASPP-application

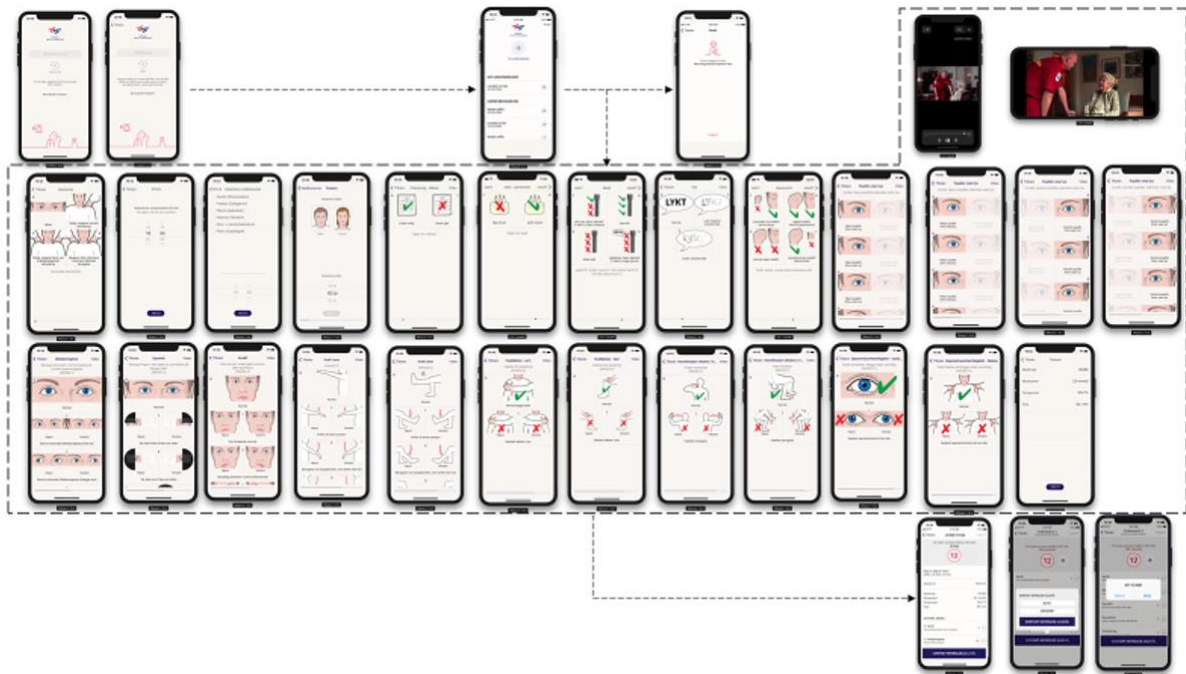

Supplement: Supplementary file 3 — Appendix S3 [file ENE-31-e16252-s003.pdf]
